# Structural basis for adhesin secretion by the outer-membrane usher in type 1 pili

**DOI:** 10.1073/pnas.2410594121

**Published:** 2024-09-24

**Authors:** Ryan M. Bitter, Maxwell I. Zimmerman, Brock T. Summers, Jerome S. Pinkner, Karen W. Dodson, Scott J. Hultgren, Peng Yuan

**Affiliations:** ^a^Department of Cell Biology and Physiology, Washington University School of Medicine, Saint Louis, MO 63110; ^b^Department of Pathology and Immunology, Washington University in St Louis, St Louis, MO 63110; ^c^Department of Biochemistry and Molecular Biophysics, Washington University in St Louis, St Louis, MO 63110; ^d^Washington University Center for Cellular Imaging, Washington University School of Medicine, Saint Louis, MO 63110; ^e^Department of Molecular Microbiology, Washington University in St Louis, St Louis, MO 63110; ^f^Center for Women’s Infectious Disease Research, Washington University in St Louis, St Louis, MO 63110; ^g^Department of Pharmacological Sciences, Icahn School of Medicine at Mount Sinai, New York, NY 10029; ^h^Department of Neuroscience, Icahn School of Medicine at Mount Sinai, New York, NY 10029

**Keywords:** type 1 pilus, pilus biogenesis, outer-membrane usher FimD, adhsein FimH

## Abstract

Gram-negative bacteria produce chaperone–usher pathway pili, which are extracellular protein fibers tipped with an adhesive protein that binds to a receptor with stereochemical specificity to determine host and tissue tropism. The outer-membrane usher protein, together with a periplasmic chaperone, assembles thousands of pilin subunits into a highly ordered pilus fiber. The tip adhesin in complex with its cognate chaperone activates the usher to allow extrusion across the outer membrane. The structural requirements to translocate the adhesin through the usher pore from the periplasm to the extracellular space remains incompletely understood. Here, we present a cryoelectron microscopy structure of a quaternary tip complex in the type 1 pilus system from *Escherichia coli*, which consists of the usher FimD, chaperone FimC, adhesin FimH, and the tip adapter FimF. In this structure, the usher FimD is caught in the act of secreting its cognate adhesin FimH. Comparison with previous structures depicting the adhesin either first entering or having completely exited the usher pore reveals remarkable structural plasticity of the two-domain adhesin during translocation. Moreover, a piliation assay demonstrated that the structural plasticity, enabled by a flexible linker between the two domains, is a prerequisite for adhesin translocation through the usher pore and thus pilus biogenesis. Overall, this study provides molecular details of adhesin translocation across the outer membrane and elucidates a unique conformational state adopted by the adhesin during stepwise secretion through the usher pore. This study elucidates fundamental aspects of FimH and usher dynamics critical in urinary tract infections and is leading to antibiotic-sparing therapeutics.

## Significance

Gram-negative bacteria produce chaperone–usher pathway (CUP) pili tipped by an adhesin, which mediates host and tissue tropisms by binding to receptors with stereochemical specificity. The structural details to translocate the adhesin through the outer-membrane usher from the periplasm to the extracellular space remain incompletely understood. We used cryoelectron microscopy to determine a quaternary complex from the type 1 pilus system depicting the FimD usher actively secreting its cognate adhesin FimH. Our study reveals the structural plasticity of the two-domain adhesin during translocation and reveals a conformation that FimH adopts during secretion. These findings reveal the molecular details of adhesin translocation across the outer membrane and provide the basis for the design of rational therapeutics.

Chaperone–usher pathway (CUP) pili are critical virulence factors in Gram-negative bacterial pathogens that mediate attachment to host and environmental surfaces. Type 1 pili expressed by uropathogenic *Escherichia coli* (UPEC) are tipped by an adhesin, FimH, that binds mannosylated glycoproteins, including those on the surface of bladder epithelial cells to facilitate attachment to and invasion of bladder superficial facet cells (1, 2). The FimH–mannose interaction is critical in the ability of UPEC to cause urinary tract infection (UTI). A FimH vaccine (3, 4) and a mannoside (5, 6) which neutralize FimH are in human clinical trials to treat and prevent UTI. Assembly of type 1 pili is facilitated by the FimC periplasmic chaperone and FimD outer-membrane (OM) usher in an ATP-independent manner (7, 8). Each pilus subunit, or pilin, adopts an incomplete immunoglobulin (Ig)-like fold lacking the carboxy-terminal β-strand thus exposing a hydrophobic groove (9, 10). To adopt its characteristic Ig-like fold, each pilin subunit receives a C-terminal β-strand, *in trans*, from the chaperone FimC. The chaperone’s G1 β-strand completes the subunit Ig-like fold in a noncanonical (parallel) manner, termed donor-strand complementation (DSC). In addition, each pilin subunit has a short (typically ~15 amino acids) N-terminal extension (Nte). At the usher, the Nte on an incoming subunit displaces the chaperone’s G1 β-strand and completes the subunit fold of the preceding subunit in a canonical (antiparallel) and energetically favored manner, termed donor-strand exchange (DSE) (9–13). The iterative process of chaperone–subunit recruitment to the usher followed by DSE between the incoming and usher-bound subunit facilitates growth of the pilus rod (*SI Appendix*, Fig. S4).

The OM usher FimD consists of five functional domains: i) a 24-stranded β-barrel translocation pore domain (TD) that spans the OM, ii) a β-sandwich plug domain (PD) that occludes the pore of the TD in the apo and closed state, iii) a periplasmic amino-terminal domain (NTD), and iv) two carboxy-terminal domains (CTD1 and CTD2) (14–17). Chaperone–subunits are first recruited to the usher NTD (18–20), particularly, disordered residues 1 to 24 that define an amino-terminal tail of the NTD (called the N-terminal tail) (16) where they interact transiently prior to handover to the usher CTDs (19, 21). Consistent with the notion that the adhesin has the highest affinity to the usher NTD (22) and localizes to the distal tip of the pilus rod, the chaperone–adhesin FimC–FimH is the first to engage with the closed apo-usher and responsible for initial usher activation. Crystal structures of the usher–chaperone–adhesin complex FimD–FimCH and the isolated FimD TD reveal that engagement of the chaperone–adhesin complex FimC–FimH on FimD triggers the release of its plug domain from the transmembrane pore to the periplasm (14). Subsequently, the β-barrel TD transitions from the apo, kidney-shaped conformation (52 × 28 Å) to a circular form (44 × 36 Å), allowing penetration of the FimH lectin domain (FimH_L_) into the pore lumen and later extrusion of folded pilin subunits (~20 to 25 Å in diameter) across the OM (14). X-ray and cryoelectron microscopy (cryo-EM) structures of FimD–FimCFGH demonstrate large coordinated movements of the five usher domains involved in catalyzing DSE interactions between neighboring subunits and extrusion of the growing fiber (15–17). These structures also show that the two-domain adhesin FimH, which consists of the lectin (FimH_L_) and pilin (FimH_P_) domains, adopts an elongated (“relaxed”) or bent (“tense”) configuration prior to and after secretion to the extracellular surface, respectively. Compared with the “relaxed” conformation, the “tense” conformation displays a ~37° decrease in angle between the FimH_L_ and FimH_P_ domains and first forms after FimH completely exits the usher pore (15). Once FimH is in the linear tip fibrillum, the conformational equilibrium between the “relaxed” and “tense” states fine-tunes binding affinity to its receptor and thus influences *E. coli* fitness in urinary tract infection (23).

Little is known about the structural requirements to fully translocate pilus subunits, specifically the two-domain adhesin, across the outer membrane through the usher. In the activated usher–chaperone–adhesin FimD–FimCH crystal structure, FimH_L_ remains within the TD pore and poised for translocation while FimH_P_ remains in DSC with the chaperone and associated with the usher CTDs in the periplasmic space (14). At this stage, the global conformation between FimH_L_ and FimH_P_ is “relaxed” and essentially identical to the conformation of FimH in the FimCH complex in the absence of the FimD usher (PDB 1QUN) (10). In the elongation complex FimD–FimCFGH, FimH is fully extruded through the TM pore as both the FimH_L_ and FimH_P_ domains have emerged to the extracellular space and adopted a “tense” conformation (15–17). Hence, these structures capture the conformational states of the adhesin prior to and after transport by the usher. Here, we report the cryo-EM structure of a quaternary complex FimD–FimCFH, in which the FimH adhesin is in a previously unseen location within the pore of the FimD usher during transport. Remarkably, during this stage in secretion, FimH adopts a third “relaxed-stretched” conformation involving both an elongation and rotation between its pilin and lectin domains. Together, these structures provide molecular insights into how the two-domain adhesin is transported across the usher pore during pilus biogenesis. The structure presented here elucidates a critical assembly intermediate that can be used to design antibiotic-sparing therapeutics to combat UPEC.

## Results and Discussion

### FimD–FimCFH Complex.

**Fig. 1.**
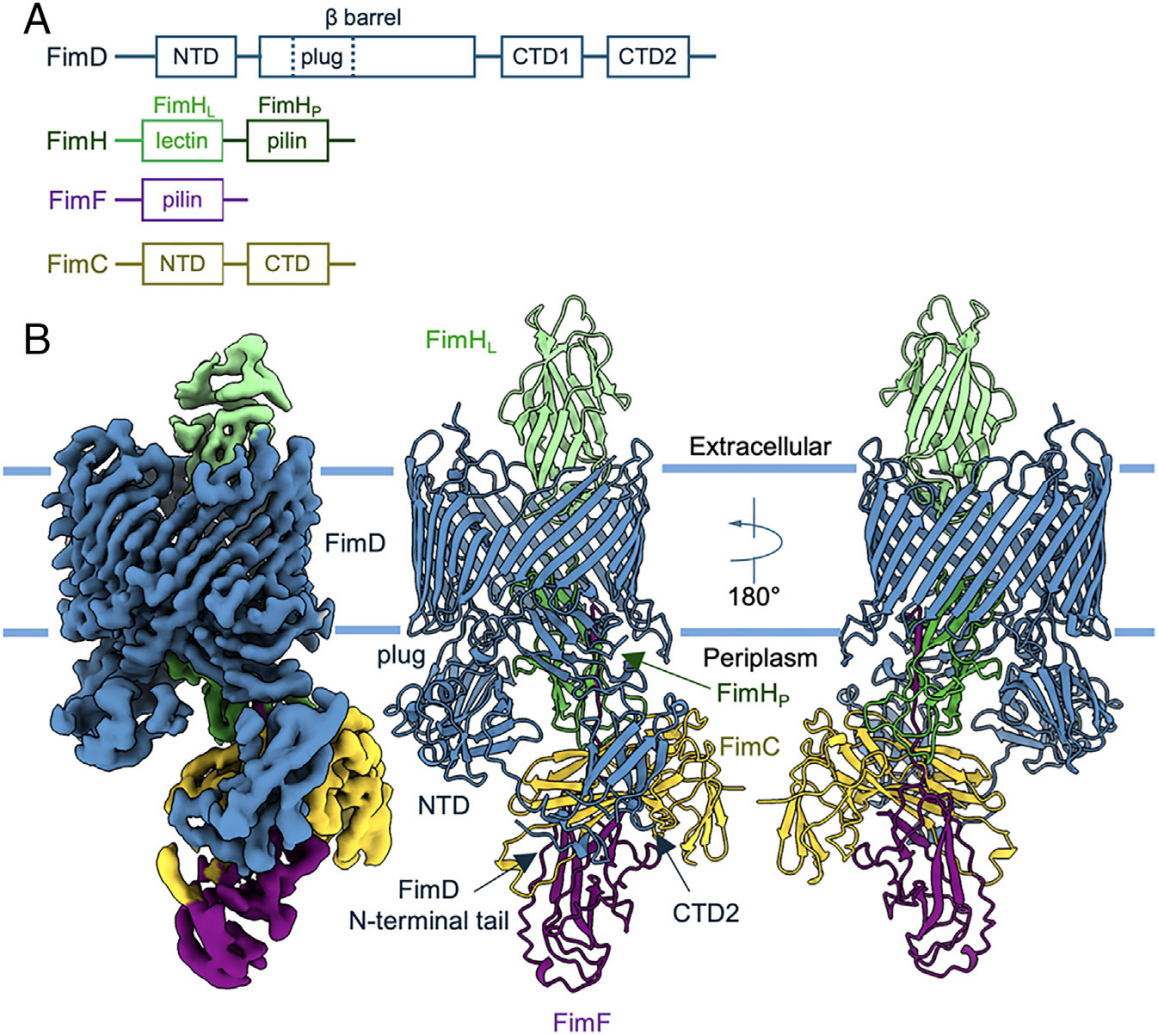
Type 1 pilus system and structure of a tip complex FimD–FimCFH. (*A*) Domains of the FimD usher, adhesin FimH, subunit FimF, and chaperone FimC. FimD consists of five functional domains: NTD, plug domain, β-barrel translocation domain, and two CTDs. FimH has a lectin domain FimH_L_ and a pilin domain FimH_P_. (*B*) Cryo-EM density and structure of FimD–FimCFH. The outer-membrane boundaries are indicated. Each subunit is uniquely colored (FimD in blue, FimH in green, FimC in yellow, and FimF in purple).

In wildtype type 1 pili, the tip adhesin FimH is followed by two tip adapters: i) FimG links FimH to FimF; and ii) FimF, which links FimGH to the FimA rod. FimD–FimC–FimF–FimG–FimH has previously been characterized (15, 16). In a system that expressed FimD–FimCFGH (16), we deleted the N-terminal extension (Nte) of FimG (FimG_ΔNte_) hypothesizing that FimG_ΔNte_ would remain bound to the N-terminal domain (NTD) of the usher prior to DSE with FimH. Deletion of the FimG N-terminal extension (FimG_ΔNte_) abolished the formation of a FimD–FimCG_ΔNte_H complex, likely because of weakened association between FimG_ΔNte_ and FimD–FimCH in the absence of DSE, which requires the Nte of FimG. The Nte of FimF is similar to the FimG Nte, and we found that we were able to form a FimD–FimCFH complex, in addition to FimD–FimCH by cryo-EM analysis (Fig. 1 *A* and *B* and *SI Appendix*, Fig. S1 and Table S1). The FimD–FimCH structure had a reported overall resolution of ~3.5 Å and was essentially identical to that published previously (14), while the FimD–FimCFH structure, at ~4.0 Å resolution, revealed a previously unreported assembly intermediate enabled by DSE between FimH and FimF. FimH, which normally undergoes DSE with FimG, has previously been shown to undergo DSE with FimF via crystallography (24) (PDB 4XOB; See *SI Appendix*, Fig. S2*C*) and in an in vitro pilus assembly system using purified subunits (25). Promiscuity between FimG and FimF for FimH is likely due to the high sequence similarity in their Ntes, in which all the hydrophobic pocket residues are identical except the third hydrophobic pocket (P3) where Ile7 of FimF can substitute Val7 of FimG. Furthermore, the pilin domains of FimF and FimG are structurally conserved, with a rmsd of 1.25 Å for Cα atoms between pilin domains (*SI Appendix*, Fig. S2*B*).

The cryo-EM structure of FimD–FimCFH represents a unique quaternary assembly intermediate, in which the usher FimD is in the process of secreting the adhesin FimH (Fig. 1B). Unlike the previously determined initiation and elongation structures, this complex reveals FimH positioned halfway in the usher pore. Here, the orientation of FimH_L_ and FimH_P_ relative to each other is even more stretched than that in the previously reported “relaxed-elongated” conformation (10, 23), while the base of the FimH_L_ closely resembles the “relaxed” FimH_L_ (*SI Appendix*, Fig. S3). Thus, we termed this conformation “relaxed-stretched.” This conformation is adopted by FimH as it spans the entire OM while retaining contact with both the periplasmic and extracellular spaces. In this model, FimH is linked to FimF through DSE between FimH and FimF ( *SI Appendix*, Fig. S2*A*). The chaperone–subunit complex FimC–FimF makes contacts with both the CTDs and N-terminal tail of the usher FimD. This usher–chaperone–subunit arrangement resembles that of one of the conformers in the elongation complexes FimD–FimCFGH, in which the chaperone–subunit complex is being transferred from the NTD to the CTDs after DSE with the preceding pilin subunit (PDB 6E14) (16).

### Stepwise Secretion of the Adhesin.

**Fig. 2.**
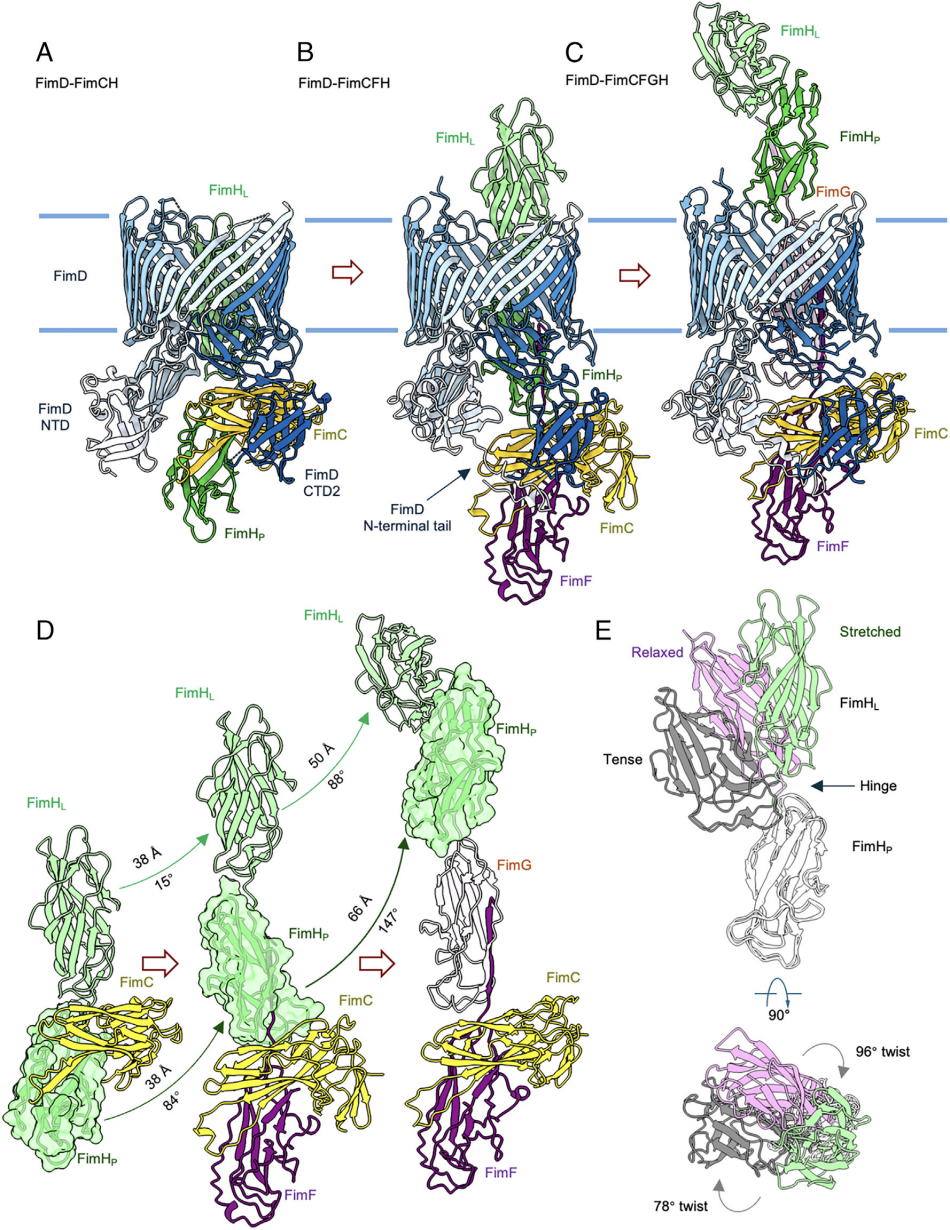
Stepwise secretion of the FimH adhesin. (*A*) Structure of the initiation complex FimD–FimCH (PDB: 3RFZ) (*B*) Structure of FimD–FimCFH. (*C*) Structure of the elongation complex FimD–FimCFGH (PDB: 6E14). The FimD usher is colored from light blue to dark blue from the amino- to the carboxy-terminus. Other subunits are similarly colored for comparison. (*D*) Domain movements in the stepwise secretion of the adhesin FimH. (*E*) Superposition of the three distinct conformations of adhesin FimH during transport. Structures are aligned on the basis of the pilin domain (FimH_P_).

The FimD–FimCFH structure, together with structures of the initiation and elongation complexes, FimD–CH and FimD–CFGH, respectively, provides detailed visualization of the stepwise secretion of the adhesin FimH through the pore of the usher (Fig. 2). Upon usher activation by the chaperone–adhesin complex and its subsequent presentation to the usher CTDs, as seen in the FimD–CH structure, the FimH_L_ domain is fully inserted into the TD pore while FimC–FimH_P_ remains anchored to the usher CTDs (Fig. 2*A*). In the absence of FimG or when the Nte of FimG is removed, FimC–FimF is expected to be recruited by the usher NTD and subsequently transferred to the CTDs. During this transfer, the N-terminal tail of the usher FimD extends from its NTD and interacts with both CTD2 and the chaperone–subunit complex FimC–FimF (Fig. 2*B*). The chaperone FimC is released from FimH_P_ as DSE occurs between FimF and FimH_P_, and FimH is pushed further into the TD pore lumen as FimC–FimF transfers to the usher CTD. With the engagement of only two pilin subunits (FimH and FimF), the FimH_P_ domain is partially inserted into the TD pore as FimF, having undergone DSE with FimH_P_, remains bound to the chaperone FimC and held by the usher NTD and CTDs in the periplasmic space. With the addition of three subunits to the growing tip, as seen in the FimD–FimCFGH elongation complex, the entire FimH_L_ and the majority of FimH_P_ have been pushed to the extracellular side of the OM (Fig. 2*C*); As such, complete extrusion of FimH into the extracellular space requires at least three pilin subunits. An intermediate between these two extremes is seen in the quaternary FimD–FimCFH complex, as FimH_L_ and FimH_P_ both partially inhabit the pore (Fig. 2*B*). As the linker between FimH_L_ and FimH_P_ is straightened within the pore lumen, only a small portion of each of the FimH_L_ and FimH_P_ domains can be accommodated by the TD pore.

The FimD–FimCFH structure reveals that during its translocation through the usher pore, the adhesin FimH undergoes drastic domain rearrangements (Fig. 2 *D* and *E*). From usher activation to adhesin secretion, the FimH_L_ domain transits from being fully embedded within the TD pore to largely emerged into the extracellular space, resulting in a translation of 38 Å and a slight rotation of 15° (Fig. 2*D*). The FimH_P_ domain is released from the periplasmic chaperone and partially inserted into the pore lumen. A comparison of these two structures indicates that FimH_L_ is oriented for translocation through the usher pore upon usher activation, and its relative orientation to the pore is largely maintained during translocation. In contrast, the pilin domain FimH_P_ undergoes substantial reorientation (a rotation of 84°) to be compatible with its residency in the TD pore (Fig. 2*D*). This indicates that the TD pore has a preferred orientation for the pilin domains that are being extruded. At the elongation stage (FimD–FimCFGH), FimH fully emerges into the extracellular space. Transition to this state requires large translation and rotation of both the FimH_L_ (50 Å/88°) and FimH_P_ (66 Å/147°) domains (Fig. 2*D*). Upon its exit from the usher pore, FimH adopts a distinct “tense” conformation while maintaining elongated conformations during insertion and secretion (Fig. 2 *D* and *E*).

Comparison of our quaternary FimD–FimCFH structure with the analogous PapC-PapDKG structure from P pili (17) shows that the adhesin of each system adopts a different position within the pore of the usher after recruitment of a second chaperone–subunit; The linker between FimH_L_ and FimH_P_ is roughly halfway across the membrane in FimD–FimCFH, whereas the analogous region of PapG in PapC-PapDKG is located at the periplasm–OM interface with PapG_P_ almost entirely in the periplasm. Interestingly, while incorporation of a third subunit to type 1 pili is sufficient to completely push the adhesin to the extracellular space (Fig. 2*C*), incorporation of a third subunit in the P pilus system results in only PapG_L_ being translocated to the extracellular side while PapG_P_ remains embedded in the usher pore (17).

A previous study (15) predicted that a combination of steep energy funnels and opposing binding surfaces position the Ig-like pilin domain of subunits at the center of the usher pore during transport. Their simulations suggested that FimH_L_ formed tighter interactions within the lumen of the usher than FimH_P_, suggesting that FimH_L_ may adopt a preferred orientation that is not the exact center. Additionally, the authors were unable to determine an apparent low-energy entry–exit pathway of FimH_L_, leading them to suggest that FimH adopts a previously uncharacterized conformation during secretion, which we believe to be the stretched-relaxed state presented here. In contrast to the transport of subunits that only contain Ig-like pilin domain, we show that FimH_L_ maintains its relative orientation within the pore throughout the first half of transport, while FimH_P_ compensates for this by undergoing substantial rotation.

Comparison of these structures also reveals remarkable structural plasticity of the two-domain adhesin FimH, which experiences three distinct conformations during its translocation across the usher pore (Fig. 2*E*). In the “tense” conformation, the lectin domain bends back toward the pilin domain, resulting in a compact conformation. In the “relaxed” conformation, FimH becomes more extended, with substantial twist between its two domains. During secretion, FimH is obligated to adopt a third, “relaxed-stretched” conformation, in which the two domains are maximally apart from each other (Fig. 2*E*). This results in a minimal cross-sectional area optimal for passing through the usher pore. In addition, the drastic twists between these two domains suggest that pilin subunits adopt preferred orientations within the usher pore, which is presumably determined by shape and surface complementation between the pilin subunit and the pore lumen. Our study has revealed a third conformational state of FimH and suggests that interconversion between conformational states of FimH is not only critical for receptor binding (23) but also for transport across the OM.

### Conformational Plasticity of FimH Is Essential for Piliation.

**Fig. 3.**
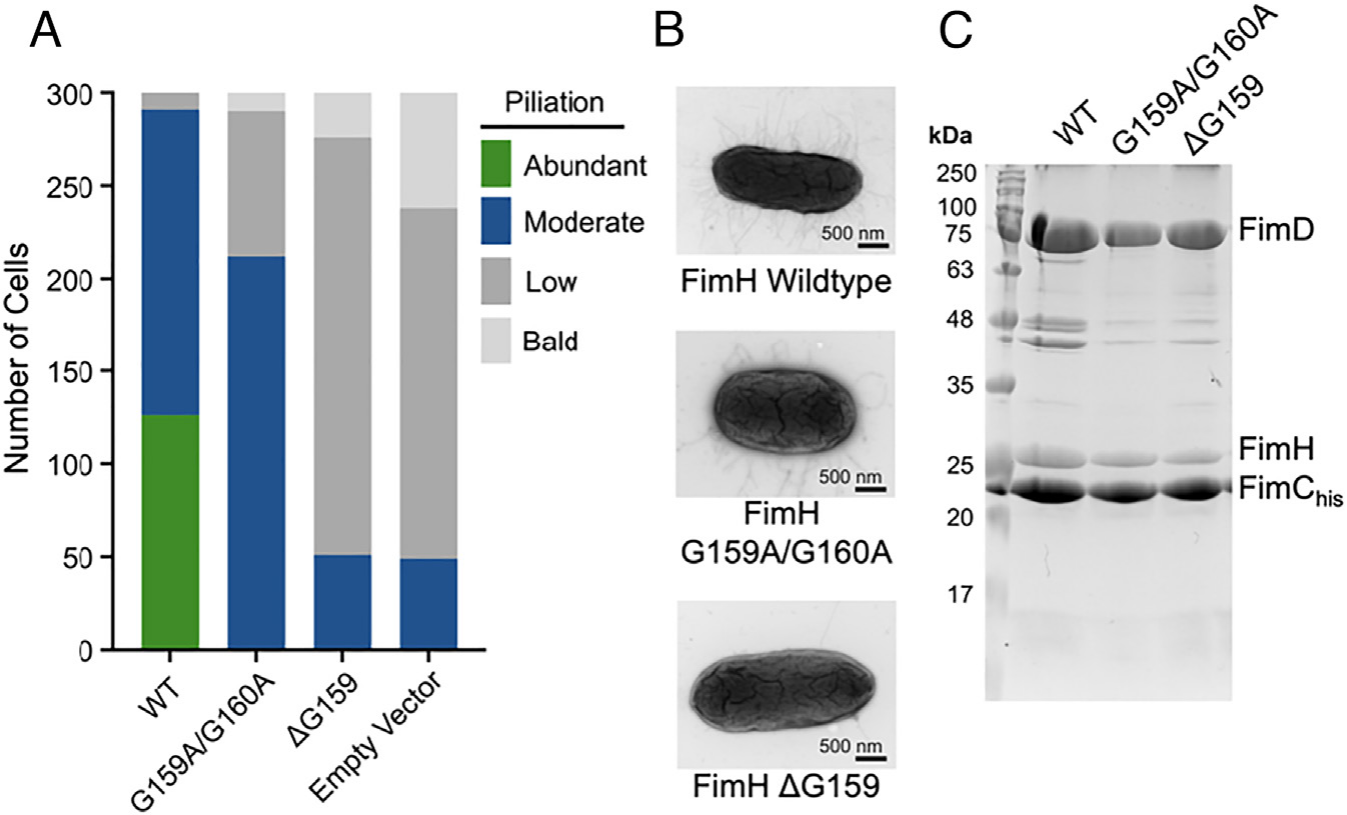
Structural plasticity between FimH lectin and pilin domains is a requirement for adhesin secretion through the FimD usher. pBAD FimC_His_–FimH with either wild-type FimH, FimH(G159A/G160A), or FimH(ΔG159) was expressed in UTI89 *E. coli* with locked-on type 1 pili with a deletion of the *fimH* gene. (*A*) Pili on the surface of the cell were counted and classified as bald (no pili), low (1 to 20 pili/cell), moderate (20 to 200 pili/cell), or abundant (>200 pili/cell). Three hundred bacterial cells were counted for each strain. (*B*) Representative negative-stain electron microscopy images showing FimC_His_–FimH_WT_ (abundant), FimC_His_–FimH(G159A/G160A) (moderate), and FimC_His_–FimH(ΔG159) (low) pili. (*C*) Outer-membrane purification of FimD–FimC_His_–FimH with the indicated mutations in FimH.

The conformational plasticity of FimH is enabled by a flexible linker connecting its two domains, which consists of two consecutive glycine (G159 and G160) residues. To assess the functional requirement of the observed conformational plasticity, we reduced the flexibility between the FimH_L_ and FimH_P_ domains by mutating the consecutive glycine residues to alanine (G159A/G160A) or removing one of the glycine residues (ΔG159) in the linker. A FimCH plasmid encoding each of these FimH linker mutants was transformed into UTI89 *E. coli* with a locked-on type 1 Δ*fimH* operon. Therefore, the FimH molecules in all type 1 pili produced by these *E. coli* cells were provided by the plasmid. Negative-stain electron microscopy was used to compare piliation across the wild-type or mutant FimH or an empty vector control. Most cells (97%) expressing the wild-type FimH produced abundant (≥100 pili/cell, 42%) or moderate pili (21 to 99 pili/cell, 55%) (Fig. 3 *A* and *B*). In contrast, cells expressing FimH (G159A/G160A) could only produce moderate pili (70.6%) (Fig. 3 *A* and *B*). FimH(ΔG159)-expressing cells produced the least numbers of pili and appeared comparable to cells without FimH expression (empty vector control, Fig. 3*A*). These results indicate that the flexible arrangement between the two adhesin domains is critical for type 1 pilus biogenesis. To rule out the possibility that these FimH mutants were not expressed or did not associate with the FimD usher, we purified the initiation complexes consisting of FimD, FimC, and wild-type or mutant FimH from the outer membranes of these cells. Both FimH(G159A/G160A) and FimH(ΔG159) retained their ability to associate with the chaperone and to engage the usher (Fig. 3*C*). Collectively, we conclude that the structural plasticity of FimH is a prerequisite for its translocation through the usher pore and thus pilus biogenesis.

### Domain Movement of the Usher.

**Fig. 4.**
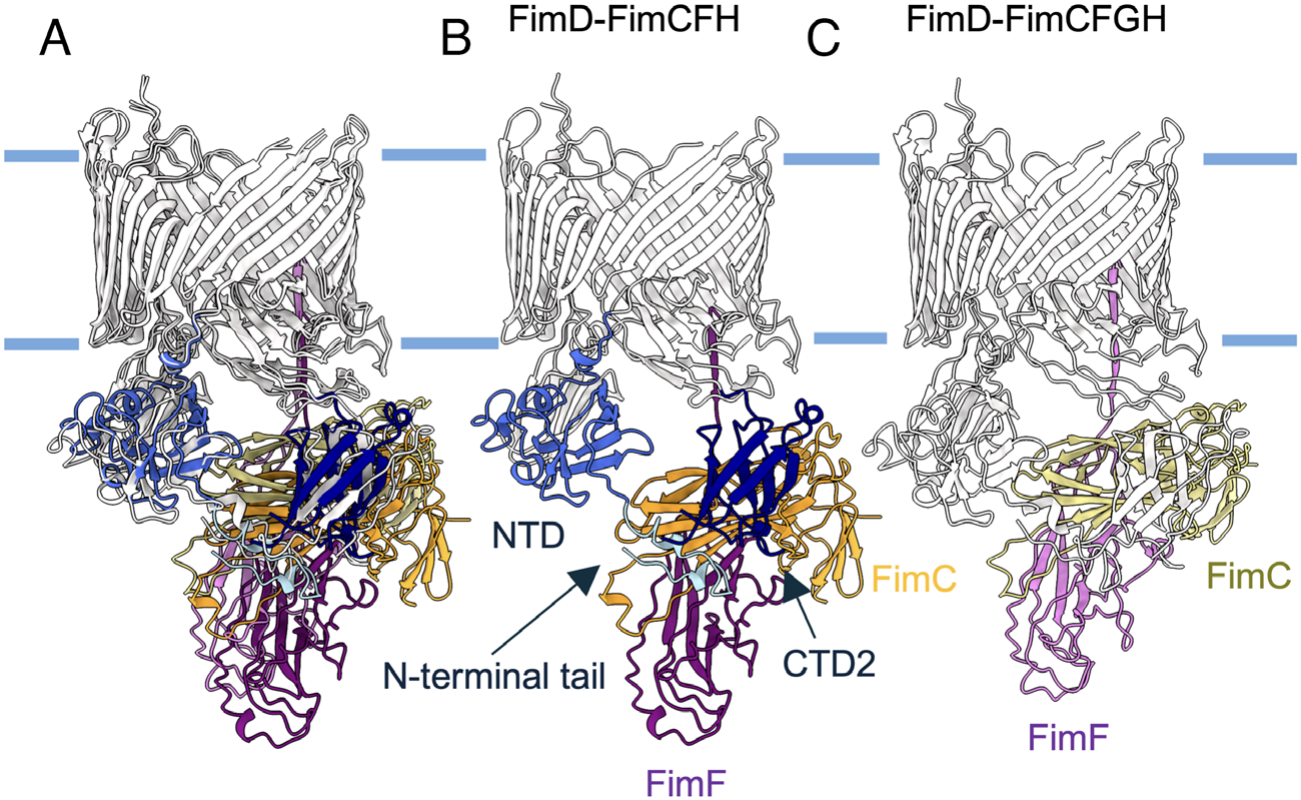
Domain movements in the usher FimD. (*A*) Superposition of FimD–FimCF structures from FimD–FimCFH and FimD–FimCFGH (PDB: 6E14). Protein components are colored in the same schemes as those in (*B*) and (*C*). (*B*) FimD–FimCF in FimD–FimCFH. (*C*) FimD–FimCF in FimD–FimCFGH.

Structures of the isolated inactive ushers and the preinitiation, initiation, and elongation complexes in the type 1 and P pilus systems have revealed large, coordinated movements of the usher domains during pilus biogenesis, such as displacement of the PD from the TD pore, transition of the TD from an oval to a circular form, and domain reorganization during subunit transfer from the NTD to CTDs (14, 16, 17). Here, our FimD–FimCFH structure uncovers further structural rearrangements of the usher domains during subunit extrusion. At this intermediate stage, the FimH_P_ domain is only partially engaged in the usher pore. This contrasts with the fully embedded FimG pilin domain in the elongation complex (Fig. 2*C*). The elongation complex displayed greater movement of the usher NTD toward the chaperone–subunit–CTD2 interface than it did in our FimD–FimCFH structure. This is consequential to the fact that the incoming subunit to be extruded, FimF, is further away from the usher pore in our structure compared with its position in the elongation complex, in which the Nte of FimF is inserted into the usher pore by 13 Å (Fig. 4). Placement of FimF in such a transition state requires coordination of the chaperone FimC as well as the usher domains, in particular, the N-terminal tail of the NTD, and CTD2. Advancement to complete adhesin secretion is accompanied by a translation and rotation of FimF (14 Å/18°), FimC (10 Å/21°), the usher NTD (3 Å/11°), N-terminal tail (7 Å/19°), and CTD2 (3 Å/19°), respectively.

## Conclusion

Pilus biogenesis is catalyzed by an OM nanomachine called the usher, which assembles and extrudes the proteinaceous fiber across its pore. This process requires exquisite choreography of the usher domains and intricate coordination of subunits. In this work, we describe the structure of the type 1 usher actively secreting the two-domain adhesive tip subunit FimH through the TD. This structure fills an important gap in our understanding of the structural dynamics of the usher and adhesin during subunit extrusion. Our study reveals further the remarkable structural plasticity of the two-domain adhesin that allows transport across the usher pore as a folded protein. Importantly, the conformational flexibility of the adhesin is a critical fitness factor in urinary tract infection. In addition, it is worth noting that in all subunits, there is a conserved length (~4 amino acids) linker between the Nte in DSE with the preceding subunit and the body of the pilin domain, suggesting that this linker spacing may also be required to allow the flexibility to the growing linear fiber to sequentially get pilin domains through the usher to the extracellular surface. Thus, our results increase our understanding of the principles of protein transport by the OM usher and potentially guide rational design of unique therapeutics targeting the adhesin molecules that are crucial for host receptor binding and bacterial infectivity.

## Materials and Methods

### Construct Design.

The starting plasmid material for the in vivo coexpression of FimD–FimCFH was kindly provided by the lab of David Thanassi (Stony Brook University) and described previously (26). pAN2 expresses the outer-membrane usher FimD without an affinity tag under an IPTG-inducible promoter. pNH237 expresses FimC–FimF–FimG–FimH with a C-terminal polyhistidine tag on FimC under an arabinose-inducible promoter. To generate a FimC_his_–FimF–FimG ΔNTE–FimH expression cassette whereby FimG does not have an N-terminal extension (FimGΔNte), pNH237 was used as the template in an inverse PCR with 5’-phosphorylated-DelNteG_Fwd primer and DelNteG_Rev primer to amplify the entire plasmid without residues 3 to 12 (VTITVNGKVV) of FimG. The PCR product was ligated, digested with DpnI, and cloned in *E. coli*. All the plasmids have been verified by sequencing.

For the piliation assay, FimC–FimH whereby FimC carried a C-terminal polyhistidine tag was subcloned from pNH237 described above. Residues in the linker region between the lectin and pilin domain of FimH were mutated via a ligation-independent cloning (LIC) technique, resulting in FimC–FimH(G159A/G160A) and FimC–FimH(ΔG159). LIC was performed by PCR amplifying the entire plasmid in two fragments with overlapping ends using primers containing the desired mutation between the annealing and overlapping sequence. For FimC–FimH(G159A/G160A), fragment 1 was amplified using G159A/G160A-Frag1-Fwd and G159A/G160A-Frag1-Rev and fragment 2 was amplified using G159A/G160A-Frag2-Fwd and G159A/G160A-Frag2-Rev (*SI Appendix*, Table S2). For FimC–FimH(ΔG159), fragment 1 was amplified using delG159-Frag1-Fwd and delG159-Frag1-Rev and fragment 2 was amplified using delG159-Frag2-Fwd and delG159-Frag2-Rev (*SI Appendix*, Table S2). In both cases, fragment 1 was approximately 9.5 kB and fragment 2 was approximately 500 bp. Fragments 1 and 2 were then mixed at a molar ratio of 1:7 (sevenfold molar excess of fragment 2), placed in a reaction with Phusion DNA polymerase, dNTPs, the Phusion High Fidelity (HF) Buffer (Thermo Scientific), and subject to incubation in the thermocycler with the following parameters: 98 °C for 40 seconds followed by 5 cycles of 98 °C for 10 seconds, 62 °C for 10 seconds and 72 °C for 5 min, and finished with 72 °C for 10 min before being held at 4 °C. LIC reactions were treated with Dpn1 overnight at 37 °C before being transformed into DH5α *E. coli* for cloning.

### FimD–FimCFH Protein Expression and Purification.

Twenty-four liters of *E. coli* strain C600 transformed with the desired plasmids were grown in Terrific Broth supplemented with 50 μg/mL kanamycin and 40 μg/mL chloramphenicol at 37 °C while shaking at 250 Rpm. When cells reached OD_600_ 0.6 to 0.8, they were induced with 0.1% arabinose and 100 µM IPTG and the temperature was decreased to 20 °C for expression overnight. After ~16 h, cells were pelleted at 4,500×*g*, flash-frozen in liquid nitrogen, and stored at −80 °C until purification.

The frozen cell pellet was thawed on ice and brought to 500 mL in ice-cold sonication buffer (50 mM Sodium Phosphate pH 7.0, 100 mM NaCl) supplemented with protease inhibitors [leupeptin (2.5 μg/ml), pepstatin A (1 μg/ml), 4-(2-aminoethyl) benzenesulfonyl fluoride hydrochloride (100 μg/ml), aprotinin (3 μg/ml), 1 mM benzamidine, and 200 μM phenylmethylsulfonyl fluoride] and deoxyribonuclease I. Cells were lysed by sonication and kept ice-cold. Unlysed cells and larger debris were pelleted at 4,500×*g* for 20 min at 4 °C, and the supernatant was treated with 1% sarkosyl, stirring at room temperature for one hour to dissolve the inner membrane. The outer membrane was pelleted at 45,000×*g* for one hour at 4 °C, and the supernatant discarded. The outer-membrane pellet was then resuspended in 150 mL solubilization buffer [1% (w/v) n-Dodecyl β-D-maltoside (DDM; Anatrace), 20 mM sodium phosphate pH 7.0, 300 mM NaCl, protease inhibitors), and stirred overnight (~16 h) at 4 °C. Solubilized protein was separated from the insoluble fraction by centrifugation for 1 h at 45,000×*g* and incubated with 3 ml of TALON metal affinity resin (Takara) overnight at 4 °C with gentle rotation. TALON resin was loaded to a column and washed with an imidazole gradient in wash buffer (20 mM sodium phosphate pH 7.0, 150 mM NaCl, 2 mM DDM). Fractions from the imidazole gradient were analyzed by SDS PAGE and coomassie blue staining, and fractions containing the target protein complex were pooled, concentrated using an Amicon Ultra-15 centrifugal filter unit (molecular weight cutoff 50 kDa) and injected into a Superose 6 Increase 10/300 gel filtration column (GE Healthcare) equilibrated in 20 mM Tris pH 8.0, 150 mM NaCl, and 0.5 mM DDM. The peak fractions were collected and concentrated to ~6 mg/ml for cryo-EM.

### Cryoelectron Microscopy (CryoEM).

Cryo-EM grids were prepared using a Vitrobot Mark IV (Thermo Fisher Scientific). Then, 3 μl of ~6 mg/ml purified protein was applied to Quantifoil R1.2/1.3 (Q350CR1.3, Electron Microscopy Sciences) holey carbon grids that were plasma cleaned for 90 s using a Solarus 950 (Gatan) with an H2/O2 mixture. After a 20 s wait, each grid was blotted for 2 s and immediately plunged into liquid ethane. Grids were clipped and loaded into a 200 kV Glacios Cryo-TEM (Thermo Fisher Scientific). Movies were recorded with a Falcon IV Direct Electron Detector (Thermo Fisher Scientific) in an automated fashion using EPU software 3.1.0 (Thermo Fisher Scientific) with a pixel size of 1.184 Å and a nominal defocus range of −1.0 to −2.4um. Data were acquired with a dose rate of 4.55 e-/Å2/s over a 10.47 s exposure time, yielding a total dose of 47.63 e-/Å2 over 45 fractions.

### Data Processing and Refinement.

Initial data processing was conducted in Cryosparc v3.3.1 (27). Seven thousand eight hundred sixty-eight movies were subject to motion correction and CTF estimation. Initial cleanup and template picking resulted in 832,428 particles, which were subject to 2D classification followed by ab initio reconstruction with three classes. All three ab initio classes underwent heterogeneous refinement followed by nonuniform refinement. At this point, it became apparent the dataset contained roughly equal amounts of particles of both FimD–FimCFH and FimD–FimCH. The cryo-EM density map of FimD–FimCH fit well with the previously determined structure (PDB 3RFZ). Density corresponding to FimD–FimCFH was used to perform another round of template picking, 2D classification, and ab initio reconstruction with 2 classes. Heterogeneous followed by nonuniform refinement on 160,422 particles resulted in a final FimD–FimCFH density map with an overall resolution of 3.99 Å. Finally, DeepEMhancer (28) was used in Cryosparc v4.2.1 to improve the overall map quality and facilitate model building.

### Model Building.

Coordinates of the FimD usher, FimC chaperone, FimF tip adapter, and FimH adhesin from PDB 3RFZ and PDB 3JWN were docked, rigid-body fit, and subjected to model building in Coot 0.9.8.7 (29). The periplasmic N-terminal domain, plug domain, and C-terminal domains 1 and 2 of the FimD usher were refined in ISOLDE (30). Iterative rounds of model adjustment in Coot followed by real-space refinement in Phenix 1.20.1 (31) were performed until outliers were minimized based upon Ramachandron plot (32, 33). The final atomic model was evaluated by MolProbity (34). Figs. were generated using ChimeraX 1.5 (35).

### Piliation Assay to Determine the Effects of FimH Plasticity on Secretion through FimD.

FimC–FimH whereby FimC carried a C-terminal polyhistidine tag was subcloned from pNH237 described above. Next, residues in the linker region between the lectin and pilin domain of FimH were mutated, resulting in FimC–FimH(G159A/G160A) and FimC–FimH(ΔG160). Plasmids were transformed into UTI89 *E. coli* that contained a locked-on type 1 operon with a deletion in *fimH*. An empty vector (pBAD33) served as the negative control. A single colony of each strain plated on LB agar supplemented with 20 μg/mL chloramphenicol was inoculated into 20 mL LB containing 0.01% arabinose in a 150 mL glass flask. Cultures were grown under stationary conditions overnight at 37 °C. The following morning (18 to 24 h), cells were pelleted and resuspended in PBS for a final OD_600_ of 1.0. Cells were then subjected to negative-stain electron microscopy for pilus counts. A total of 300 bacterial cells were counted for each strain, and piliation on cells was classified as bald (no pili), low (1 to 20 pili/cell), moderate (21 to 99 pili/cell), or abundant (100 ≥ pili/cell).

## Supplementary Material

Appendix 01 (PDF)

Movie S1. FimH adhesin translocation through the FimD β-barrel pore. Video generated by sequentially morphing FimH from the activation (FimD-FimCH; PDB 3RFZ), secretion (FimD-FimCFH, present study), and elongation (FimD-FimCFGH; PDB 6E14) models after first aligning the FimD usher in all models.

## Data Availability

Protein Structure data have been deposited in PDB (9BOG) (36).
