# Supplementary material for: Structural basis for adhesin secretion by the outer-membrane usher in type 1 pili
Source: Proc Natl Acad Sci U S A. 2024 Sep 24;121(40):e2410594121. doi: 10.1073/pnas.2410594121 (PMC11459180; doi:10.1073/pnas.2410594121)
Supplement: Supplementary file 1 — Appendix 01 (PDF) [file pnas.2410594121.sapp.pdf]

Supporting Information for

Structural basis for adhesin secretion by the outer-membrane usher in type 1 pili

Ryan M. Bitter, Max Zimmerman, Brock T. Summers, Jerome S. Pinkner, Karen W. Dodson, Scott J. Hultgren, Peng Yuan

Corresponding authors: Scott J. Hultgren and Peng Yuan
Email: hultgren@wustl.edu, peng.yuan@mssm.edu

This PDF file includes:

Figures S1 to S4
Tables S1 to S2

Other supporting materials for this manuscript include the following:

Movie S1

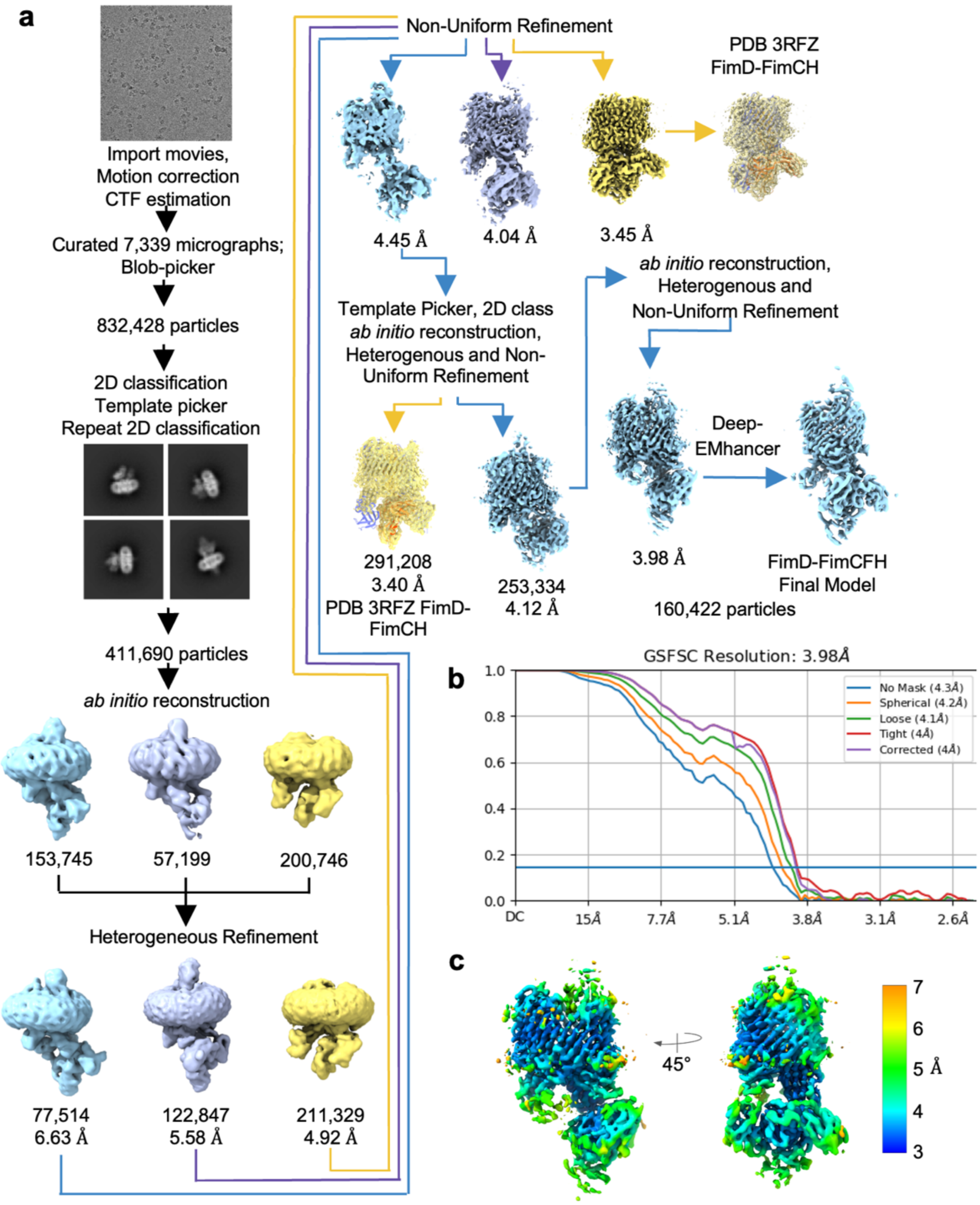

Fig. S1. Cryo-EM data processing flowchart for FimD-FimCFH. a, Image processing and density refinement. b, Fourier shell correlation calculated in cryoSPARC between half maps. c, Local resolution estimation.

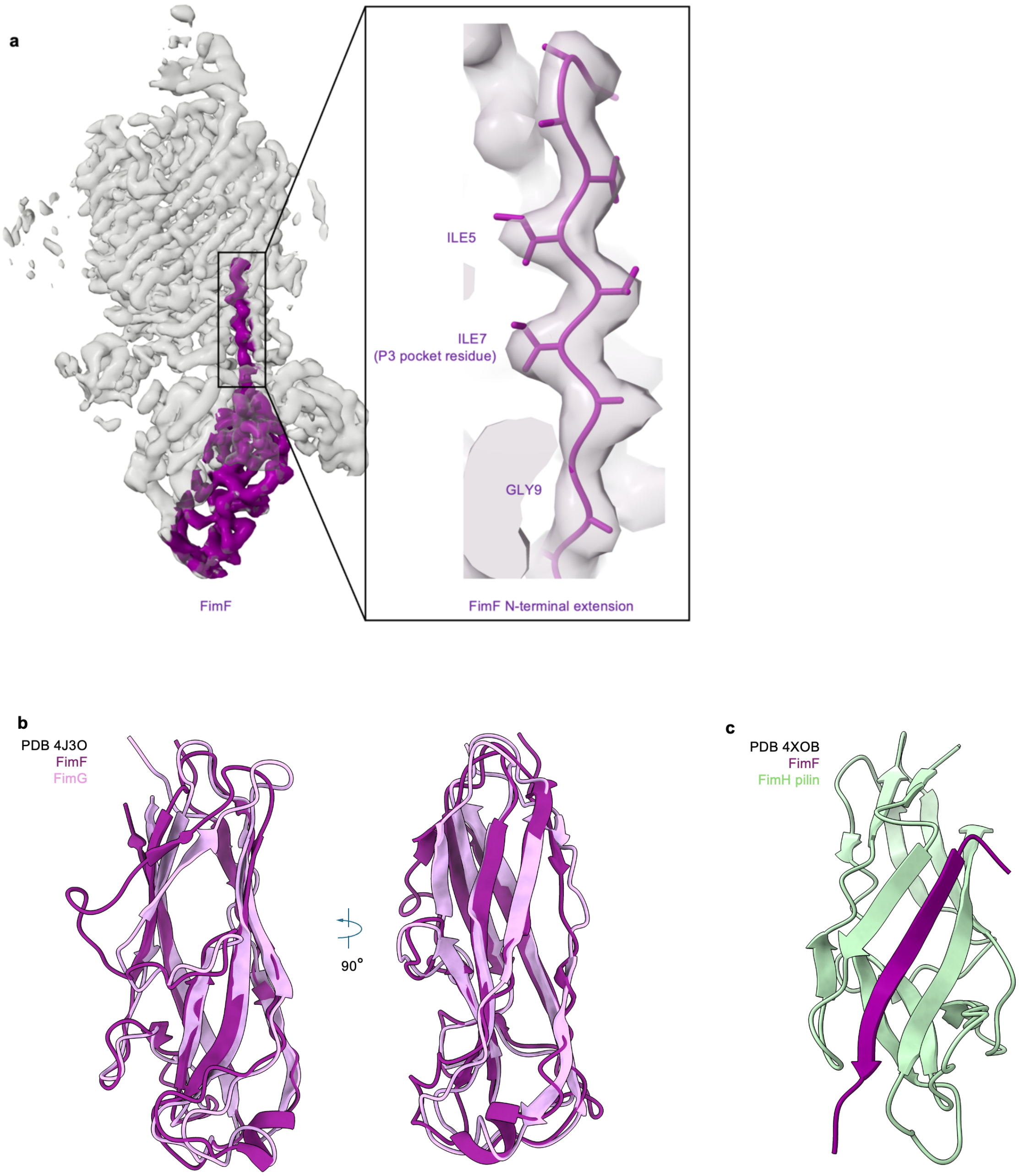

Fig. S2. DSE between FimF and FimH. a, Cryo-EM density of Nte of FimF. b, Structural alignment of pilin domains between FimF and FimG (R.M.S.D of 1.25 Å for Cα atoms between pilin domains) from PDB 4J3O. c, Crystal structure of FimF Nte (purple) in DSE with FimH (green) from PDB 4XOB.

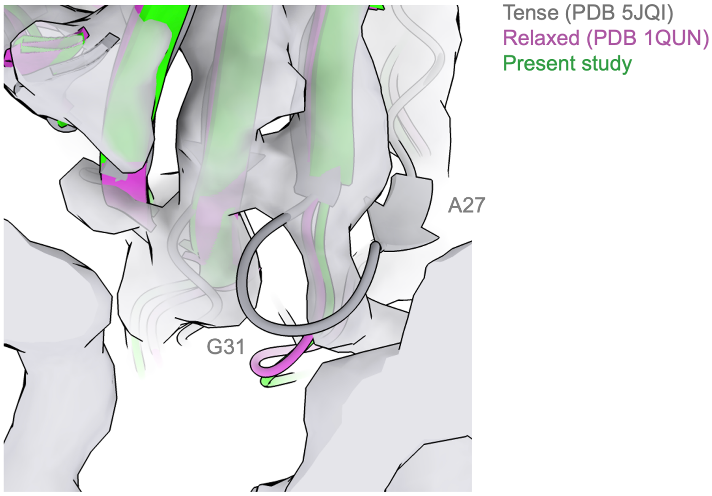

Fig. S3. Comparison of previously published models of the 'relaxed' (PDB 1QUN) and 'tense' (PDB 5JQI) conformations of $FimH_L$ docked into our cryo-EM density. Depicted here is the region surrounding A27, which in combination with a mutation at V163 in the pilin domain, shift the conformational equilibrium of FimH towards the relaxed state[1]. G31 is labeled to orient the reader. Since our cryo-EM density better fits the 'relaxed' conformation of $FimH_L$ in this region, we have called our new conformation "stretched-relaxed" to describe both the global FimH conformation (i.e. 'stretched' between lectin and pilin domains) and the $FimH_L$ conformation (i.e. 'relaxed' lectin domain).

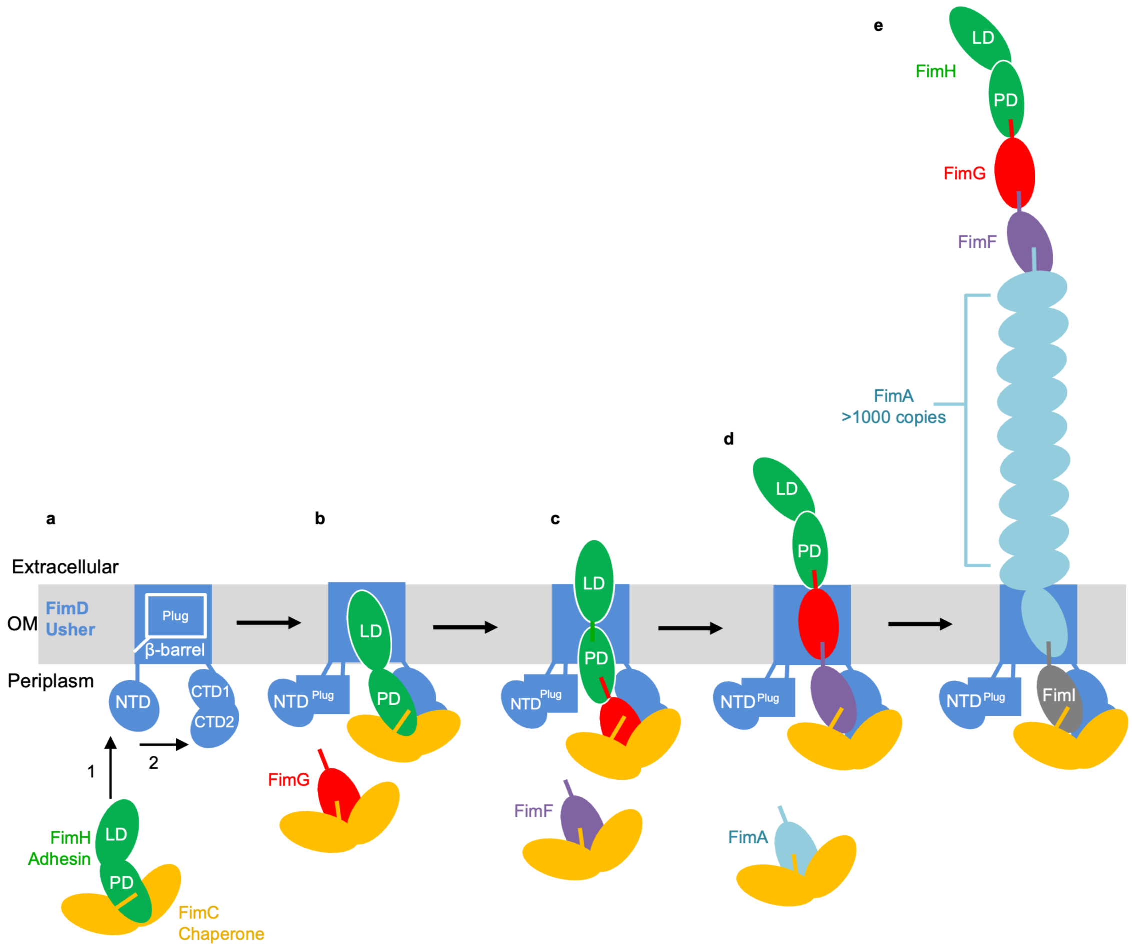

Supplemental Figure S4. Simplified schematic of Type 1 pilus assembly at the OM usher. **a,** The chaperone-adhesin (FimCH) is targeted to the outer membrane (OM) usher (FimD). FimCH is believed to first engage the FimD N-terminal domain (NTD) prior to handover to the usher C-terminal domain (CTD). **b,** FimCH binding to FimD results in displacement of the FimD plug domain (PD) from the β-barrel pore to the periplasm. After activation and PD displacement, FimCH is held by the usher CTDs. **c,** Subsequent chaperone-subunits are targeted to the FimD NTD, undergo donor-strand exchange (DSE) with the proceeding subunit, and are anchored to the usher CTD. **d,** FimA, which composes the pilus rod, is targeted to the usher **e,** Thousands of FimA subunits form the helix rod. Pilus growth is terminated by FimI.

Table S1. cryoEM data collection and model statistics

	FimDCFH
**Data collection and processing**	
Magnification	120k
Voltage (kV)	200 kV
Electron exposure (e–/Å2)	47.63
Defocus range (μm)	-1.0 to -2.4
Pixel size (Å)	1.184
Symmetry imposed	C1
Initial particle images (no.)	832,428
Final particle images (no.)	160,422
Map resolution (Å)	3.99
FSC threshold	0.143
Map resolution range (Å)	3.0 - 6.0
**Refinement**	
Model resolution (Å)	4.2
FSC threshold	0.5
Model composition	
Nonhydrogen atoms	9,371
Protein residues	1,438
Ligands	0
B factors (Å^2)	
Protein	60.74
Ligand	N/A
R.m.s. deviations	
Bond lengths (Å)	0.002
Bond angles (°)	0.535
Validation	
MolProbity score	1.58
Clash score	6.86
Poor rotamers (%)	0.44
Ramachandran plot	
Favored (%)	96.77
Allowed (%)	3.23
Disallowed (%)	0

Table S2. Nucleotide sequence of PCR primers used in this study.

**Primer Name**	**Sequence (5'-3')**
DelNteG_Fwd	(5'-phosphorylation)-ATCGGCTGCCTGTATCG
DelNteG_Rev	GCCAAACCGTGTACGG
G159A/G160A-Frag1-Fwd	CCAATAATGATGTGGTGGTGCCTACTGCCGCCTGCGATGTT TCTGCTCGTGATGTCACCG
G159A/G160A-Frag1-Rev	CCGCCAATAGGGATAGCGGTACCATTGGC
G159A/G160A-Frag2-Fwd	GCCAATGGTACCGCTATCCCTATTGGCGG
G159A/G160A-Frag2-Rev	CGGTGACATCACGAGCAGAAACATCGCAGGCGGCAGTAGG CACCACCACATCATTATTGG
delG159-Frag1-Fwd	CGCCAATAATGATGTGGTGGTGCCTACTGGCTGCGATGTTT CTGCTCGTGATGTCACCG
delG159-Frag1-Rev	CAGGCGAATGACCAGGCATTTACCGACCAGC
delG159-Frag2-Fwd	GCTGGTCGGTAAATGCCTGGTCATTCGCCTG
delG159-Frag2-Rev	CGGTGACATCACGAGCAGAAACATCGCAGCCAGTAGGCAC CACCACATCATTATTGGCG

Movie S1 (separate file)

FimH adhesin translocation through the FimD β-barrel pore. Video generated by sequentially morphing FimH from the activation (FimD-FimCH; PDB 3RFZ), secretion (FimD-FimCFH, present study), and elongation (FimD-FimCFGH; PDB 6E14) models after first aligning the FimD usher in all models.

SI References:

1. Kalas V, Pinkner JS, Hannan TJ, Hibbing ME, Dodson KW, Holehouse AS, Zhang H, Tolia NH, Gross ML, Pappu RV, Janetka J, Hultgren SJ. Evolutionary fine-tuning of conformational ensembles in FimH during host-pathogen interactions. Sci Adv. 2017 Feb 10;3(2):e1601944.
